# Electroacupuncture for chemotherapy-induced hemifacial spasm: a case report

**DOI:** 10.3389/fmed.2026.1700524

**Published:** 2026-01-26

**Authors:** Xin Tan, Wenlong Bao, Dehou Deng, Chao Lu, Weiji Chen

**Affiliations:** 1 Department of Emergency Medicine, Tongde Hospital of Zhejiang Province, Hangzhou, China; 2 Department of Traditional Chinese Medicine, Zhejiang Cancer Hospital, Hangzhou, China; 3 Department of Acupuncture and Moxibustion, The Third Affiliated Hospital of Zhejiang Chinese Medical University (Zhongshan Hospital of Zhejiang Province), Hangzhou, China

**Keywords:** case report, cervical cancer, chemotherapy, electroacupuncture, hemifacial spasm

## Abstract

**Introduction:**

Hemifacial spasm (HFS) is a neurological disorder characterized by involuntary, paroxysmal twitching of facial muscles, primarily presenting as sudden and recurrent contractions on one side of the face. This case report described the therapeutic effect of electroacupuncture (EA) in a cervical cancer patient who developed HFS following chemotherapy.

**Case description:**

A 60-year-old female patient with cervical cancer developed severe involuntary twitching of the right facial muscles following chemotherapy, which significantly impaired her daily activities. The patient was diagnosed with HFS and was introduced to the acupuncture department to receive EA treatment. Following 15 sessions of EA treatment, the patient’s HFS symptoms improved significantly, with complete resolution of facial muscle twitching.

**Conclusion:**

The case suggests that EA may be an effective alternative treatment for chemotherapy-induced HFS.

## 1 Introduction

Hemifacial spasm (HFS) is a neurological disorder characterized by involuntary twitching of facial muscles, primarily presenting as sudden, recurrent, and subjectively uncontrollable contractions on one side of the face (1, 2). The etiology of HFS is complex and multifactorial, thought to be associated with facial nerve irritation or compression, and closely linked to factors such as fatigue and mental–emotional states (3). Previous studies have suggested that acupuncture may be a potential therapeutic option for HFS (4, 5). Electroacupuncture (EA) integrates traditional acupuncture with modern electrical stimulation technology. A growing body of clinical evidence supports the utility of EA in mitigating a spectrum of chemotherapy-related adverse effects, including but not limited to chemotherapy-induced peripheral neuropathy (CIPN), nausea/vomiting, and pain (6–8). These findings underscore EA’s potential as a non-pharmacological adjunctive intervention for managing chemotherapy-related toxicities in oncology settings. However, there is a paucity of literature regarding the efficacy of EA in cancer patients with chemotherapy-induced HFS. Herein, we present a case of a 60-year-old female cervical cancer patient with severe HFS who attained complete remission after 100 Hz EA treatment. This report complies with the CARE extension guideline for acupuncture case reports (9).

## 2 Case presentation

### 2.1 Clinical presentation

A 60-year-old female with cervical cancer was admitted to the Acupuncture Department of Zhejiang Cancer Hospital for management of “persistent right facial muscle twitching for over 6 months.” The patient had been diagnosed with cervical cancer in September 2021, for which she received radical radiotherapy combined with concurrent cisplatin-based chemotherapy. During a follow-up examination in March 2023, lung metastases were detected, and she was diagnosed with post-radiotherapy cervical cancer with lung metastases, prompting further radiation therapy. In July 2024, follow-up evaluations revealed progressive lung metastases, along with new metastases to the left kidney and lymph nodes in the right supraclavicular fossa, mediastinum, and hilar regions. Following a multidisciplinary team (MDT) consultation at Zhejiang Cancer Hospital, she initiated Taxane plus Carboplatin (TC) regimen chemotherapy on September 3, 2024, combined with immunotherapy using Xindilimumab.

After completing four consecutive cycles of chemotherapy (by November 19, 2024), the patient developed involuntary twitching of the right facial muscles, accompanied by numbness and weakness in the extremities. During the subsequent chemotherapy interval, her facial spasm symptoms showed slight alleviation; however, they exacerbated significantly following the fifth cycle. By January 6, 2025, she had completed 6 cycles of TC regimen chemotherapy combined with immunotherapy. At this point, her right facial muscle twitching had worsened, severely disrupting sleep. Additionally, the numbness and fatigue in her extremities also worsened, with the numbness assessed as grade 2 according to the Common Terminology Criteria for Adverse Events (CTCAE). Over the subsequent months, the patient sought treatment at multiple facilities to alleviate her “facial muscle twitching” symptoms. The enhanced cranial MRI showed no obvious abnormality. So brain metastasis was excluded. Her attending physician prescribed oral methylcobalamin tablets, but no improvement was observed. She came to the Acupuncture Department of Zhejiang Cancer Hospital on April 16, 2025, for seek further treatment.

### 2.2 Diagnosis assessment

The patient exhibited frequent involuntary contractions of the right facial muscles, particularly involving the periorbital region. Involuntary eye movement was observed during eye closure (Supplementary Video 1). Spasm severity was graded III according to the Cohen criteria (10). She complained that the facial muscle twitching persisted even during nighttime sleep, severely impairing her quality of life. Additionally, she experienced numbness and weakness in her hands and feet. Assessment of facial sensory nerve function showed no abnormalities. Based on the aforementioned clinical manifestations, the patient was diagnosed with HFS, cervical cancer with multiple metastases, CIPN, and cancer-related fatigue.

## 3 Intervention and outcome

**Figure 1:**
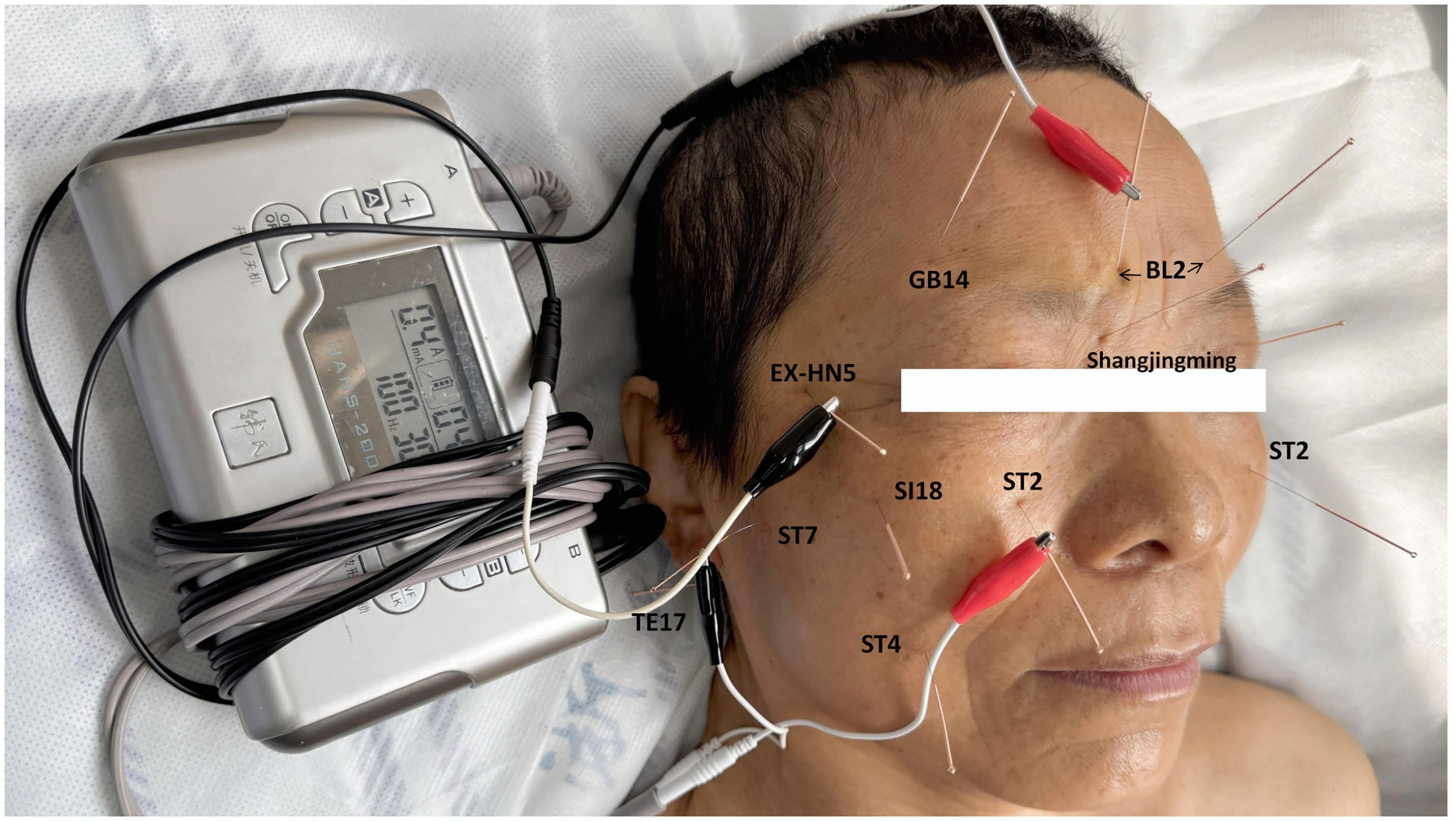
Treatment scenario including acupoint selection and EA instrument parameter settings.

At the initial consultation on April 16, 2025, a treatment plan centered on EA therapy was formulated for the patient. The primary facial acupoints selected were: bilateral Cunzhu (BL2), bilateral Taiyang (EX-HN5), bilateral Sibai (ST2), as well as Yangbai (GB14), Shangjingming (an extra point), Quanliao (SI18), Dicang (ST4), Xiaguan (ST7), and Yifeng (TE17) on the affected side. The locations of these acupoints (as presented in Table 1) adhered to the World Health Organization (WHO) standards (11). Disposable acupuncture needles (0.18 mm × 25 mm) were used. The patient was placed in a supine position, and the acupoint areas were sterilized. During needle insertion, the patient was instructed to close her eyes. All needles were inserted to a depth of approximately 15–23 mm, aiming to elicit a local sensation of soreness and distension in the facial muscles. BL2 was paired with EX-HN5, and ST2 was paired with ST7 for EA stimulation using a Han’s Acupoint Nerve Stimulator (HANS-200E, Nanjing Jisheng Co., Ltd). The EA parameters were set as follows: frequency of 100 Hz and intensity adjusted to the patient’s tolerance. For each session, all acupoints involved were stimulated with acupuncture for 30 min, but only BL2 and EX-HN5, ST2 and ST7 were treated with 100 Hz electroacupuncture stimulation while retaining needles, with the same stimulation time of 30 min. The acupoint selection and EA application are illustrated in Figure 1. All acupuncture and EA interventions were performed by a licensed acupuncturist with 8 years of work experience.

**Table 1:** Location of acupoints.

Acupoints	Location
Cuanzhu (BL2, both side)	Located in the depression on the medial eye of eyebrow, on the supraorbital notch.
Taiyang (EX-HN5, both side)	Located in the depression about one finger-breadth posterior to the midpoint between the lateral end of the eyebrow and the outer canthus.
Sibai (ST2, both side)	Located directly below the pupil, at the depression of the lower edge of the eye socket.
Yangbai (GB14, right side)	Located directly above the pupil, 1 cun above the brow arch (eyebrow bone).
Shangjingming (Extraodinary acupoint, right side)	About 0.2 cun above the corner of the inner eye’s canthus.
Quanliao (SI18, right side)	Located in the depression at the lower edge of the cheekbones, with the outer canthus (outer corner of the eye) straight down.
Dicang (ST4, right side)	Located outside the corner of the mouth, and directly below the pupil position.
Xiaguan (ST7, right side)	Located in front of the facial ear, in the depression formed by the zygomatic arch and mandibular notch.
Yifeng (TE17, right side)	Located behind the earlobe, in the depression between the mastoid process and the mandibular angle.

**Figure 2:**
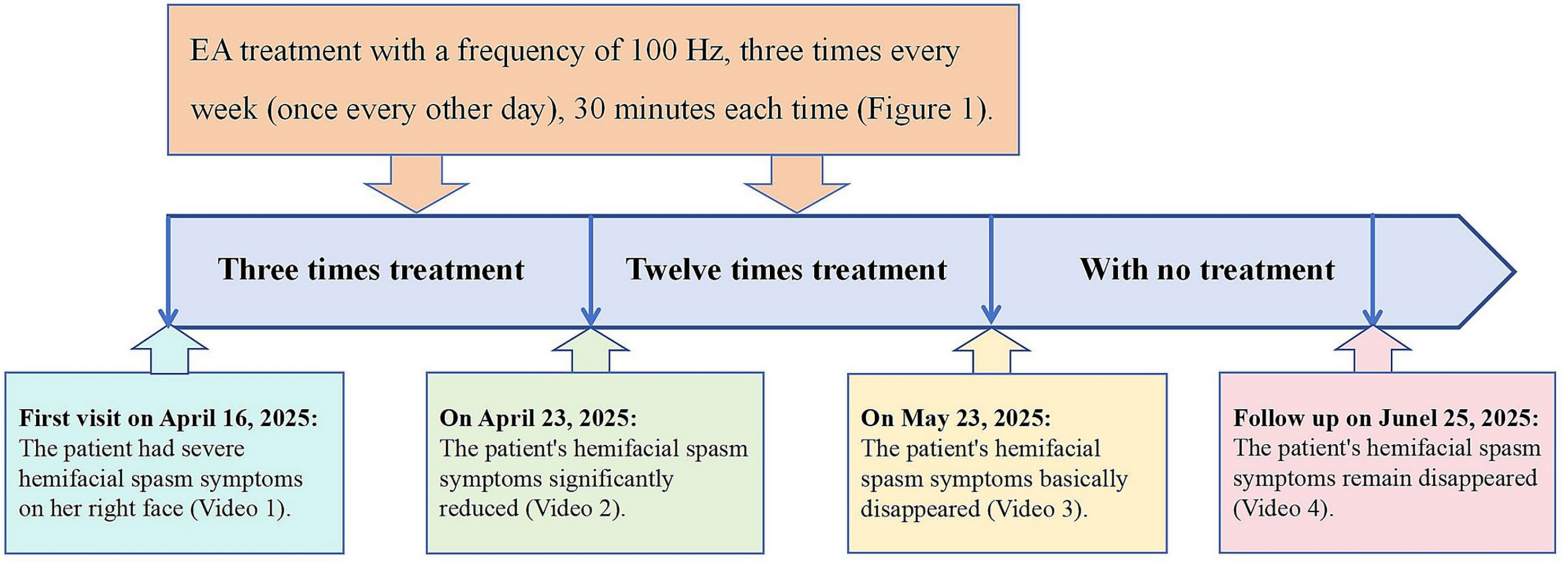
Treatment timeline.

Before the first treatment, the patient exhibited severe HFS symptoms (Supplementary Video 1), and spasm severity was graded III according to the Cohen criteria. Following three treatment sessions (by April 23, 2025), the patient was pleasantly surprised to say that her right facial muscle beat symptoms were significantly improved (Supplementary Video 2), and spasm severity was graded II according to the Cohen criteria. She then continued acupuncture treatment for approximately one month, completing a total of 12 sessions. By May 23, 2025, the right facial muscle twitching had essentially disappeared (Supplementary Video 3), and spasm severity was graded I according to the Cohen criteria. The patient stated that facial spasm no longer impacted her daily life, thus ending the treatment. During a follow-up on June 25, 2025, the patient remained free of facial twitching, with no recurrence observed (Supplementary Video 4). No adverse events occurred during the treatment process. The patient had also experienced a marked improvement in sleep quality, with concurrent alleviation of fatigue symptoms. Nevertheless, mild to moderate numbness persists in the extremities. The treatment timeline is depicted in Figure 2.

## 4 Discussion

HFS is a prevalent neurological disorder characterized by involuntary, paroxysmal twitching of unilateral facial muscles. In severe cases, it can impair patients’ facial expressions, visual function, speech, and daily activities (1, 2). Clinically, the symptoms typically initiate from the orbicularis oculi muscle and progressively extend downward to involve the zygomatic muscles, oral commissure, and even the platysma (12). Initially, patients may only experience occasional eyelid twitching; however, as the disease progresses, the frequency and intensity of twitching gradually increase, particularly during emotional stress, fatigue, or periods of focused attention (13). In modern medical theory, the pathogenesis of facial spasm is often associated with abnormal stimulation of the facial nerve. For instance, factors such as vascular compression, tumors, or inflammation that affect the facial nerve root can lead to heightened excitability of the facial nerve, thereby triggering abnormal contractions of facial muscles (14, 15). In the present case, despite the presence of multiple systemic metastases in this cervical cancer patient, there is no evidence of metastasis to the central nervous system or direct involvement of the facial nerve. The patient has a history of radiotherapy for lung metastatic malignancy. Notably, the radiotherapy field does not encompass regions that would compromise facial nerve function; moreover, the temporal interval between radiotherapy administration and the onset of HFS is substantial, precluding a causal association between the two. The patient received combined chemotherapy with paclitaxel and carboplatin. Following chemotherapy administration, she developed obvious CIPN symptoms, which were assessed as graded 2 according to the CTCAE. Notably, her HFS symptoms also emerged post-chemotherapy and showed alleviation during the inter-chemotherapy intervals. Therefore, it is hypothesized that her facial spasm might be associated with the neurotoxicity of chemotherapeutic agents.

Peripheral neurotoxicity represents a common adverse effect of numerous chemotherapeutic drugs, especially in chemotherapeutic drugs of platinum, taxanes, and vinblastine (16, 17). It is generally acknowledged that the neurotoxicity of chemotherapeutic agents predominantly affects the extremities, manifesting as numbness, pain, or other paresthesias in the hands and feet, and may even induce motor nerve symptoms such as limb weakness and convulsions. In contrast, their impact on cranial nerves is relatively rare. However, previous studies have documented that chemotherapeutic drugs can cause oculomotor nerve dysfunction (18) and may even affect the central nervous system, leading to manifestations such as “chemotherapy brain” or “chemotherapy fog” (19, 20) resulting in memory loss and even cognitive dysfunction. Therefore, it can be considered that the side effects of chemotherapy drugs may be a risk factor for central nervous system diseases and the possibility of affecting the cranial nerve. However, a notable limitation of the present study was the absence of facial muscle electromyography testing in this patient. To more precisely evaluate the severity of facial muscle spasms, we applied the Cohen criteria, which served to mitigate this methodological shortcoming.

Acupuncture and EA have been widely reported as effective interventions for HFS, with substantial therapeutic benefits documented in the literature (21–23). Clinical studies have demonstrated that acupuncture or EA stimulation at specific facial acupoints can regulate facial nerve function, enhance local blood circulation, and mitigate facial muscle spasms (24). It is reported that acupuncture-related therapy is effective in treating HFS, particularly in patients with a shorter disease duration and milder symptoms (25, 26). Even for individuals with a prolonged disease course, severe conditions, or suboptimal responses to other treatments, acupuncture can still alleviate symptoms to a certain extent and improve quality of life. It is generally accepted that for limb weakness and muscle paralysis, low-frequency EA or EA with alternating high and low-frequency waveforms is preferable (27, 28). In contrast, high-frequency EA is more appropriate for managing muscle spastic disorders (29, 30). Our team previously investigated the therapeutic effect of EA with varying frequencies for CIPN. We found that low-frequency EA (2 Hz) confers distinct advantages in ameliorating sensory nerve symptoms, such as numbness in hands and feet. Low-frequency EA (2 Hz) or alternating high–low frequency waveforms (2/100 Hz) both demonstrate notable potential for improving motor nerve symptoms characterized by limb asthenia, weakness, and reduced muscle strength. Conversely, for motor nerve symptoms manifesting as muscular spasms, clonus, or elevated muscle tone, high-frequency EA (100 Hz) exhibits superior therapeutic potential (6, 31). In the present case, we selected 100 Hz EA to stimulate specific facial acupoints, which yielded excellent therapeutic outcomes. In this case, the patient presents with unilateral HFS, which manifests as facial sensory asymmetry between the two sides. Isolated acupuncture of the affected side may exacerbate facial muscular tension and spasms. In contrast, bilateral facial acupuncture can restore a sense of symmetry in the patient, thereby facilitating the alleviation of muscular spasms. This approach aligns with the empirical principle of “treating left-sided disorders via the right side” in traditional Chinese acupuncture theory. Thus, low-intensity acupuncture stimulation on the unaffected side may be administered to promote bilateral sensory-motor balance. Following over one month of EA treatment, her HFS symptoms completely resolved, and her sleep quality improved significantly. The patient also stated that her overall mood has significantly improved.

## 5 Conclusion

This case report is innovative because it shows that EA is effective for treating chemotherapy-induced HFS. Due to the limitations of the individual case, large randomized clinical trials with adequate observation and follow-up are warranted to demonstrate the curative effects of EA.

## Data Availability

The datasets presented in this study can be found in online repositories. The names of the repository/repositories and accession number(s) can be found in the article/Supplementary material.

## Supplementary material

SUPPLEMENTARY VIDEO 1 Before treatment.

SUPPLEMENTARY VIDEO 2 After 3 times of EA treatment.

SUPPLEMENTARY VIDEO 3 After 15 times of EA treatment.

SUPPLEMENTARY VIDEO 4 Follow-up (one month after the last treatment).
