# Supplementary material for: Electroacupuncture for chemotherapy-induced hemifacial spasm: a case report
Source: Front Med (Lausanne). 2026 Jan 26;13:1700524. doi: 10.3389/fmed.2026.1700524 (PMC12883351; doi:10.3389/fmed.2026.1700524)
Supplement: Supplementary file 5 [file Data_Sheet_1.PDF]

Table 1

CARE for acupuncture checklist

Domain	Item no.	Item	CARE	Reported on page
Title	1	The words 'case report' or 'case series' should be in the title along with the area of focus.	The words 'case report' should be in the title along with the area of focus	P1
Keywords	2	Two to five keywords—include 'case report' and 'acupuncture'.	Two to five keywords that identify areas covered in this case report	P1
Abstract	3a	Introduction—What is unique about this case? What does it add to the medical literature?	Introduction—What is unique about this case? What does it add to the medical literature?	P1
	3b	The main symptoms of the patient and the important clinical findings should be described *both in conventional medicine and traditional medicine*, if applicable.	The main symptoms of the patient and the important clinical findings	P1
	3c	Define the main diagnoses and therapeutic interventions. Provide *both conventional medicine diagnosis and TM diagnosis with TM pattern, if applicable. Interventions add a selection of points, operation, treatment procedures and auxiliary intervention measures.*	The main diagnoses, therapeutic interventions and outcomes	P1
	3d	Conclusion of this case.	Conclusion—What are the main 'take-away' lessons from this case?	P1
Introduction	4	Summarising the background of the case and the reason why this case is unique or meaningful with references.	One or two paragraphs summarising why this case is unique with references	P2
Patient information	5a	De-identified demographic information and other patient-specific information.	De-identified demographic information and other patient-specific information	P2
	5b	Main concerns and symptoms of the patient *and/or TM-related symptoms should be included (eg, night sweats, afternoon tidal fever and aversion to heat (or cold))*, if applicable.	Main concerns and symptoms of the patient	P2-3
	5c	Medical, family and psychosocial history including relevant genetic information (also see timeline), if applicable.	Medical, family and psychosocial history including relevant genetic information (also see timeline)	P2-3
	5d	Relevant past interventions and their outcomes.	Relevant past interventions and their outcomes	P2-3
Clinical findings	6	Describe the relevant physical examination (PE) and other significant clinical findings, *especially TM signs on tongues and pulse, eg, string pulse, red tongue, pale face*, if applicable.	Describe the relevant PE and other significant clinical findings	P3
Timeline	7	Patient's history organised in a timeline.	Important information from the patient's history organised as a timeline	P3
Diagnostic assessment	8a	Diagnostic methods shall be described *both/either in conventional medicine method and/or TM syndrome differentiation (eg, eight principles differentiation, Zang-fu organs differentiation and six meridians differentiation).*	Diagnostic methods (such as PE, laboratory testing, imaging, surveys)	P3
	8b	Diagnostic reasoning including *the analysis process of conventional medicine and/or TM differentiation based on its relevant symptoms and signs.*	Diagnostic challenges (such as access, financial or cultural)	P3
	8c	Prognostic characteristics (such as staging in oncology) where applicable.	Diagnostic reasoning including other diagnoses considered	P3
Therapeutic intervention	9a	*Style/principle of acupuncture treatment (eg, Traditional Chinese Medicine, Korean Medicine, Japanese Medicine, Western medical acupuncture), type of acupuncture (eg, electroacupuncture, fire acupuncture, ear acupuncture or scalp acupuncture) and combined therapy of acupuncture or other interventions (eg, moxibustion, herbs, lifestyle advice, Western medicine).*	Types of intervention (such as pharmacological, surgical, preventive, self-care)	P4
	9b	*Acupuncture prescription (eg, type of needle, selection of points, operation, depth and angle of insertion, direction of twitch, the manipulation method (involving thrusting, lifting and rotating techniques), intensity of these manipulation methods, response sought, needle retention time, treatment procedures and auxiliary intervention measures).*	Administration of intervention (such as dosage, strength, duration)	P4
	9c	*Any changes in acupuncture prescription (eg, acupuncture points, manipulation, needle types) during the treatment should be clearly explained, with its TM theory* if applicable.	Changes in intervention (with rationale)	Not applicable
	9d	Practitioner background regarding acupuncture (eg, qualification-training hours, work place, practice history, education background, other relevant experience).		P4
Follow-up and outcomes	10a	Clinician and patient-assessed outcomes (when appropriate).	Clinician and patient-assessed outcomes (when appropriate)	P4-5
	10b	Describe important follow-up diagnostic *and acupuncture treatment, especially the changes of TM syndrome differentiation* and other test results if applicable.	Important follow-up diagnostic and other test results	P4-5
	10c	Intervention adherence and tolerability (How was this assessed?).	Intervention adherence and tolerability (How was this assessed?)	P4-5
	10d	Adverse and unanticipated events. *Specific adverse events of acupuncture*, eg, fainting during acupuncture treatment, needle breakage, stuck needle should be reported if applicable.	Adverse and unanticipated events	P5
Discussion	11a	Discussion of the strengths and limitations in your approach to this case. *Description of the potential advantages and disadvantages of acupuncture compared with conventional treatment, if applicable.*	Discussion of the strengths and limitations in your approach to this case	P6
	11b	Discussion of the relevant medical literature.	Discussion of the relevant medical literature	P5-7
	11c	The rationale for conclusions (including assessment of possible causes). *Please be cautious in drawing general conclusions in unique cases.*	The rationale for conclusions (including assessment of possible causes)	P6-7
	11d	The primary 'take-away' lessons of this case report.	The primary 'take-away' lessons of this case report	P7
Patient perspective	12	*The retrospective survey of acupuncture (eg, therapeutic efficacy, accessibility, advantages, cost and individualised treatment experience) should be emphasised.*	When appropriate, the patient should share their perspective on the treatments they received	P7
Ethics and informed consent	13	Please provide *ethics* and informed consent if requested.	Did the patient give informed consent? Please provide if requested	P8

- The changes on CARE have been marked in italics and bold.
- TM, traditional medicine.
